# An automated micro solid phase extraction gas chromatography–mass spectrometry (μSPE-GC–MS) detection method for geosmin and 2-methylisoborneol in drinking water

**DOI:** 10.1038/s41598-023-28543-x

**Published:** 2023-01-31

**Authors:** R. L. Bristow, A. Haworth-Duff, I. S. Young, P. Myers, M. R. Hampson, J. Williams, S. Maher

**Affiliations:** 1 Department of Electrical Engineering and Electronics, University of Liverpool, Liverpool, UK; 2 Institute of Life Course and Medical Sciences, University of Liverpool, Liverpool, UK; 3 Department of Chemistry, University of Liverpool, Liverpool, UK; 4 United Utilities, Lingley Mere Business Park, Warrington, UK

**Keywords:** Analytical chemistry, Mass spectrometry, Environmental sciences

## Abstract

Geosmin and 2-methylisoborneol (2-MIB) are amongst the most common earthy and musty taste and odour (T&O) compounds found in drinking water. With low odour threshold detection limits below 10 ng L^−1^, and the complexity of raw water matrices, these two compounds provide a significant challenge for water companies globally. In this research, for the first time, a novel and fully automated micro-solid phase-extraction (μSPE) method coupled with gas chromatography (GC)–mass spectrometry (MS) has been developed for the detection of geosmin and 2-MIB for drinking water analysis. The new automated method described herein is environmentally friendly requiring low raw water sample volumes, of 25 mL, and only 50 μL of elution solvent. Our μSPE-GC–MS method exhibits excellent linearity for both compounds (R^2^ > 0.999) and low limits of detection of 2.0 ng L^−1^ and 4.3 ng L^−1^ for geosmin and 2-MIB, respectively. The method showed excellent recovery rates (95.1–100.1%) and good precision (RSD < 7%) in raw sample matrices. Our approach is fully automated onto a robotic workstation which can be readily integrated into a laboratory workflow for routine water analysis. Furthermore, the method has excellent potential to be incorporated within a portable system for onsite analysis.

## Introduction

Taste and odour (T&O) compounds provide a major challenge for water companies globally. Earthy and musty T&O compounds, primarily geosmin and 2-methylisoborneol (2-MIB), are perceived by customers as an indication of poor drinking water quality. The presence of geosmin and 2-MIB, at low nanogram per litre concentrations, often leads to consumer complaints. Therefore, it is necessary that these unpleasant compounds be regularly monitored, and treated where needed, before reaching the consumer. However, the extremely low detection threshold limits for geosmin and 2-MIB, typically below 10ng L^−1^, coupled with the complex raw sample matrices, provides a significant challenge for the water industry^1–5^. Additionally, there is a growing desire for the global water industry to move towards ‘greener’ and more efficient methods that are automated, low-cost with minimal environmental impact.

At present, several different methods exist for the detection of T&O compounds in drinking water. Most methods reported in the literature usually require some form of pre-conditioning step followed by gas chromatography—mass spectrometry (GC–MS)^6,7^. There are several pre-conditioning steps available, including: closed-loop stripping analysis (CLSA)^8–10^, solid phase microextraction (SPME)^11–13^, stir-bar sorptive extraction (SBSE)^14,15^, purge and trap (P&T)^16,17^, liquid–liquid extraction (LLE)^18,19^ and liquid–liquid microextraction (LLME)^20,21^. However, most of these methods are highly labour intensive, complex, time consuming, expensive, difficult to automate and/or require high solvent volumes^6,7^. One of the most widely used pre-conditioning methods for T & O analysis is solid-phase extraction (SPE)^22,23^.

SPE has successfully been used to extract and pre-concentrate T&O compounds from drinking water, as a pre-conditioning step prior to GC–MS analysis/quantification. Table 1 gives a summary of the methods, and key results, found within the literature for geosmin and 2-MIB detection for drinking water analysis using SPE. Low nanogram per litre detection limits are achievable, ranging from 0.1 to 5.5 ng L^−1^. However, most of the SPE methods described in Table 1 require large sample volumes, of around 100–1000 mL, to achieve the necessary sensitivity. These large sample volumes are usually loaded over open-ended SPE cartridges, resulting in long sample loading times. Additionally, many of the methods require extensive conditioning and washing of cartridges before and after runs. Likewise, many require additional post-extraction stages, such as Wright, et al.^24^ centrifuging their extractant at 1000 rpm and Ikai, et al.^25^ using a headspace extraction at 70 °C for 30 min prior to GC–MS detection. Wright, et al.^24^ and Kim, et al.^26^ also require SPE cartridge drying times at various stages in their methods to remove any residual sample or wash solvent.

**Table 1:** Summary and comparison of five detection methods for geosmin and 2-MIB from drinking water using solid phase extraction (SPE).

	Ikai et al.^25^	Ma et al.^27^	Sun et al.^28^	Wright et al.^24^	Kim et al.^26^	This study
Cartridge type	C18	LC C18	C18	IRIS Plus 6 cc	PBX	Hydrophilic C18
Cartridge volume	500 mg	500 mg	500 mg	200 mg	20 mg	3.7 ± 0.2 mg
Particle size	37–55 μm		40–60 μm	25–35 μm		3 μm
Sample volume	1 L	200 mL	1 L	1 L	100 mL	25 mL
Pre-conditioning solvent	1. 10 mL of ethanol 2. 10 mL of methanol 3. 10 mL of water	1. 10 mL of ethyl acetate 2. 5 mL of methanol 3. 10 mL of water	5 mL of methanol	1. 2 mL of ethyl acetate 2. 4 mL of methanol 3. 4 mL of milli-Q water	1. 2 mL of water × 2 2. 5 min drying	1. 100 μL of 2-propanol 2. 500 μL of water
Sample flow rate	15 mL min^−1^	5 mL min^−1^	5 mL min^−1^	30 mL min^−1^		1 mL min^−1^
Elution	1. 1 mL of ethanol 2. 10 mL of water 3. Final elution volume adjusted to 25 mL with water	2 mL of ethyl acetate	3 mL of methanol	1. 10 min air drying 2. 400 μL of ethyl acetate (2 min contact time) 3. 1 min air drying 4. 700 μL of ethyl acetate (4 min contact time)	1. 1 mL of acetone:hexane (3:7) 2. 2 μL of acetone (with 0.2% polyethylene glycol 200 and 10 μg mL^−1^ phenanthrene-d10) 3. Final volume adjusted to 1 mL with acetone:hexane (3:7)	50 μL of 2-propanol
Post-SPE extraction	Headspace (HS) analysis of elution: 12.5 mL of SPE enriched sample in a 25 mL HS vial. 6 ng of *n*-decyl chloride and 4 g of NaCl were added to the samples and shaken Extraction at 70 °C for 30 min	1. 10 µL internal standard of 1-chlorotcane added 2. Dried by sodium sulphate		1. Centrifuge 1 min at 1000 rpm 2. Ethyl acetate layer removed for analysis		
LOD	GSM: 0.1 ng L^−1^ 2-MIB: 0.1 ng L^−1^		GSM: 0.5 ng L^−1^ 2-MIB: 0.5 ng L^−1^	GSM: 0.9 ng L^−1^ 2-MIB: 5.5 ng L^−1^	GSM: 0.6 ng L^−1^ 2-MIB: 0.9 ng L^−1^	GSM: 2.0 ng L^−1^ 2-MIB: 4.3 ng L^−1^
Linearity (*R*^2^)			GSM: 0.9905 2-MIB: 0.9923	GSM: 0.993 2-MIB: 0.993	GSM: > 0.999 2-MIB: > 0.999	GSM: 0.9998 2-MIB: 0.9994
Recovery % (concentration measured at)	GSM: 104% (1 ng L^−1^) 2-MIB: 115% (1 ng L^−1^)	Pure water GSM: 62.4% (50 ng L^−1^) 2-MIB: 44.2% (50 ng L^−1^) Tap water GSM: 37.4% (200 ng L^−1^) Source water GSM: 40.4% (200 ng L^−1^)	GSM: 103% (5 ng L^−1^) 2-MIB: 98.5% (5 ng L^−1^)	Pure water GSM: 90% (25.9 ng L^−1^) 2-MIB: 95% (27.1 ng L^−1^)	Tap water GSM: 85.3% (5 ng L^−1^) 2-MIB: 61.3% (5 ng L^−1^) Raw water GSM: 104.7% (5 ng L^−1^) 2-MIB: 104.4% (5 ng L^−1^)	Raw water (reservoir) GSM: 97.8–100.0% (10–200 ng L^−1^) 2-MIB: 96.2–98.2% (10–200 ng L^−1^) Raw water (river) GSM: 96.9–97.7% (10–200 ng L^−1^) 2-MIB: 95.1–96.7% (10–200 ng L^−1^)
Relative standard deviation (RSD) % (concentration measured at)	GSM: 6.1% (1 ng L^−1^) 2-MIB: 7.5% (1 ng L^−1^)	Pure water GSM: 22.1% (50 ng L^−1^) 2-MIB: 20.1% (50 ng L^−1^) Tap water GSM: 15.5% (200 ng L^−1^) Source water GSM: 19.5% (200 ng L^−1^)	GSM: 3.7% (5 ng L^−1^) 2-MIB: 1.6% (5 ng L^−1^)	Pure water GSM: 8.5% (25.9 ng L^−1^) 2-MIB: 10.9% (27.1 ng L^−1^)	Pure water GSM: 4.5% (5 ng L^−1^) 2-MIB: 6.9% (5 ng L^−1^) Raw water GSM: 14.2% (5 ng L^−1^) 2-MIB: 14.6% (5 ng L^−1^)	Raw water (reservoir) GSM: 2.6–4.4% (10–200 ng L^−1^) 2-MIB: 1.2–4.0% (10–200 ng L^−1^) Raw water (river) GSM: 1.8–3.0% (10–200 ng L^−1^) 2-MIB: 1.9–7.0% (10–200 ng L^−1^)

Geosmin (GSM), 2-methylisoborneol (2-MIB).

As a consequence of running these high sample volumes, often with complex and time-consuming method steps, it invariably results in long extraction times for geosmin and 2-MIB detection. Routine water industry laboratories often require large numbers of samples to be analysed daily. Therefore, using one of the aforementioned SPE methods would be highly time-consuming and very labour intensive for commercial analysis, where rapid turnaround times are highly desirable.

The evolution of SPE to miniaturised micro-solid phase extraction (µSPE) systems, with sorbent masses decreasing from grams (g) to micro-grams (µg) and elution volumes from milli-litres (mL) to micro-litres (µL), has developed over the last 50 years^29^. μSPE techniques have successfully been used for the detection of several different analytes from a variety of liquids, including: blood^30^, urine^31,32^ and water^33,34^. This has significantly reduced the sample and elution volumes required. Furthermore, μSPE has increased the scientific scope for semi-automated or fully automated methodologies, reducing the need for large scale laboratory setups or full-time technical staffing, as well as eliminating operator errors. Thus, improving the quality of data whilst decreasing overall costs and turnaround times for results. Additionally, the μSPE cartridges have a significantly reduced particle diameter of around 2–3 μm, compared to conventional SPE cartridges at around 40–60 μm, significantly increasing the surface area to volume ratio and thus the overall extraction efficiency.

Similar μSPE devices to those used in this study using a one-way loading valve (see “Materials and methods”, section “μSPE cartridges and robotic workstation”) can be found in the literature, such as the work by Alexandrou, et al.^33^ and Porto-Figueira, et al.^35^. Porto-Figueira, et al.^35^ investigated the extraction of phenolic compounds, particularly catechins and quercetin derivatives, from teas comparing five different sorbent bed materials against five elution solvents. They were able to obtain comparable limits of detection (LODs), 3.5–16.9 μg L^−1^, to that obtained from conventional SPE cartridges, and demonstrated excellent recoveries of 83.0–100% for all phenolic compounds. Sample volumes were kept extremely low at 200 μL and elution volumes at 50 μL. Similarly, Alexandrou, et al.^33^ showed excellent performance using μSPE design for the extraction of trihalomethanes (THMs) from water. THMS are produced as disinfectant by-products from water treatment and are classed as emerging contaminants of concern for the industry. They demonstrated a significant enhancement in the recoveries of THMs from a small 200 μL water sample in under 2 min, with only 50 μL of elution solvent. Conventional SPE extraction methods, to provide a comparable extraction efficiency for THMs in water, typically require upwards of 120 min to condition 100 mL of sample and 1–10 mL of elution solvent.

In this present investigation, we hypothesised that an analytical method incorporating a one-way μSPE can significantly advance the analysis of geosmin and 2-MIB for drinking water analysis, to allow a greener and more efficient methodology, with reduced costs and increased throughput. To our knowledge, no research has been conducted using μSPE for geosmin and 2-MIB extraction/analysis from water. Herein we have developed, optimised, and evaluated a new μSPE-GC–MS method, including testing with raw water samples.

## Materials and experimental method

### Chemicals and reagents

Geosmin (> 97%) was purchased from Sigma-Aldrich Ltd (Dorset, UK). 2-MIB (97.7%) was purchased from Chemservice, Inc (Merseyside, UK). cis-decahydro-1-napthol (Sigma-Aldrich Ltd, UK) was used as an internal standard. Ethanol, 2-propanol, methanol, and n-hexane organic solvents were all HPLC or GC grade (Sigma-Aldrich Ltd, UK). Samples were prepared using ultra-pure type 1 water. Raw water samples were collected by United Utilities water and wastewater services in the northwest of England (Warrington, UK).

### μSPE cartridges and robotic workstation

**Figure 1:**
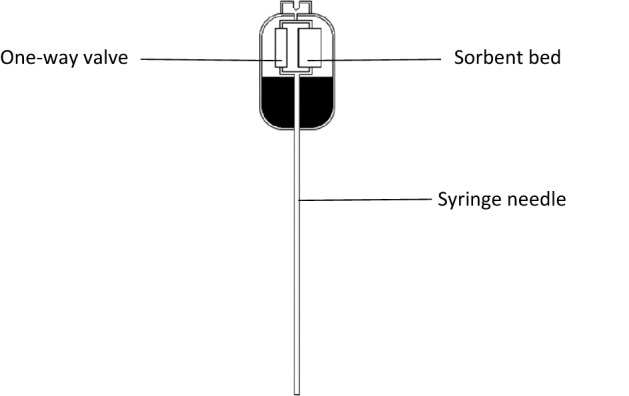
Illustrative sketch of the internal components of the μSPE sample preparation cartridges.

Six different types of μSPE cartridges were purchased from ePrep Pty Ltd (Melbourne, Australia); a C4 (particle size of 3 µm and porosity of 120 Å), C8 (particle size of 3 µm and porosity of 120 Å), C18 (particle size of 3 µm and porosity of 120 Å), hydrophilic C18 (particle size of 3 µm and porosity of 120 Å), PS/DVB (polystyrene/divinylbenzene) (particle size of 3 µm and porosity of 300 Å) and a bare silica (particle size of 3 µm and porosity of 120 Å). Figure 1 shows an anatomical sketch of the of μSPE cartridge containing the sorbent bed and a pressure driven fluoroelastomer one-way valve which prevents the sorbent bed from being back filled.

An ePrep® robotic analytical syringe sample preparation workstation was used for all method development and testing. The workstation provides a benchtop laboratory station which can fully automate sample preparation; it is controlled by programmable workflows using axis rapid workflow development software (ePrep Pty). Software interfacing was performed using a Microsoft Surface Pro tablet. Analysis was conducted using 500 μL ePrep, eZy- connect syringes. The equipment provided a fully automated sample preparation method for geosmin and 2-MIB extraction.

### μSPE sample extraction procedure

Workflows were created for the μSPE sample extractions on the workstation, a summary overview of the final method is described below (also see Supplementary Information, Fig. S1). Prior to each sample extraction the internal standard, cis-decahydro-1-napthol, was spiked into the water samples from pre-made stock solutions in methanol to produce a final concentration of 200 ng L^−1^. For the extraction process the hydrophilic C18 μSPE cartridge was conditioned with 100 μL of 2-propanol and then equilibrated with 500 μL of type 1 water at a flow rate of 1000 μL min^−1^. These stages ensure that the sorbent beds are fully activated and that any bound analytes, from previous samples, are removed. Additionally, the type 1 water wash will remove any remaining elution solvent from the sorbent bed to increase analyte binding. Samples were extracted through successive syringe loading cycles, culminating in a total sample volume of 25 mL, to ensure sufficient sensitivity for geosmin and 2-MIB detection requirements. Subsequently, cartridges were washed with 500 μL of type 1 ultrapure water at 1000 μL min^−1^ to ensure that any residue or unbound compounds were removed from the sorbent bed. The remaining bound analytes were eluted, using 50 μL of 2-propanol at 100 μL min^−1^, into a 2 mL autosampler glass vial containing a 400 μL vial insert. A graphical summary of this process can be seen in Figure S2 in the supplementary information. Extracted samples were then analysed via GC–MS detection which is described in further detail in the following section.

### Chromatographic and MS conditions

An Agilent 7890B GC coupled to a 5977A MSD system was used for analysis. The GC–MS contained an Agilent 5190-2293: 900 μL (single taper, ultra-inert) liner. Solvent injection volumes were calculated based upon the inlet liner volume, the inlet temperature, the inlet pressure, and the solvents properties [boiling point (°C), density (g cm^−3^) and molecular weight (amu)]. Vapour volumes were kept below 75% of the inlet maximum capacity and were as follows: *n*-hexane (2.7 μL), methanol (0.8 μL), ethanol (1.2 μL) and 2-propanol (1.4 μL). The GC–MS operational parameters are summarised in Table 2.

**Table 2:** Operating conditions for GC–MS used in this study.

Operating parameters for GC/MS	
Column dimensions	30 m × 0.25 mm × 0.25 μm
Carrier gas	Hydrogen
Oven temperature programme	50 °C (held for 2 min), 10 °C/min to 280 °C (held for 1 min)
Column flow	1.6 mL min^−1^
Injection mode	Splitless
Inlet pressure	5.04 psi
Inlet temperature	280 °C
Ionisation	Electron ionisation (EI)
Ionisation energy	70 eV
Auxiliary temperature	280 °C
Source Temperature	230 °C
Solvent delay	3.0 min
Scan range	50–500
SIM	95 *m/z* (quant.), 107 *m/z* (qual.) for 2-MIB, 112 *m/z* (quant.), 125 *m/z* (qual.) for geosmin, 136 *m/z* for cis-decahydro-1-naphthol

## Method development

### μSPE cartridge and elution solvent selection

**Figure 2:**
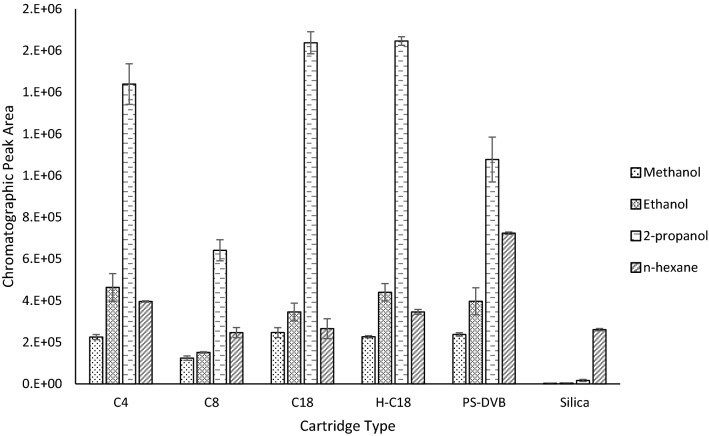
Performance of geosmin across four solvents (methanol, ethanol, 2-propanol, and n-hexane) for six different μSPE cartridge types (C4, C8, C18, hydrophilic C18 (H-C18), PS/DVB and Silica).

**Figure 3:**
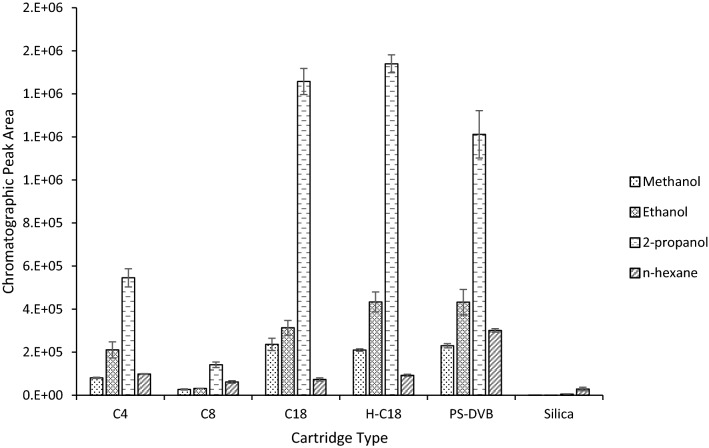
Performance of 2-MIB across four solvents (methanol, ethanol, 2-propanol, and n-hexane) for six different μSPE cartridge types (C4, C8, C18, hydrophilic C18 (H-C18), PS/DVB, Silica).

The performance of six different μSPE cartridges, in combination with four elution solvents, were compared for both geosmin and 2-MIB extraction from Type 1 water. The μSPE cartridge types were: C4 (3 µm/120 Å), C8 (3 µm/120 Å), C18 (3 µm/120 Å), hydrophilic C18 (3 µm/120 Å), PS/DVB (polystyrene/divinylbenzene) (3 µm/300 Å) and silica (3 µm/120 Å). The four elution solvents used were methanol, ethanol, 2-propanol, and n-hexane. The comparative performance for these different cartridge and solvent combinations can be seen in Figs. 2 and 3. Samples (n = 3) were produced from spiking methanol stock solutions of geosmin and 2-MIB into Type 1 water samples. For each case final concentrations of 100 μg L^−1^ and sample volumes of 8 mL were used to ensure sufficient analyte levels could be seen for all cartridge/solvent combinations for a visible comparison of the extraction performance during optimisation. Elution volumes were set to 200 μL to ensure complete elution of all analytes and to ensure consistency across all solvent types.

The C4, C8, C18, hydrophilic C18 and PS/DVB cartridges are all considered surface modified silica cartridges, with either a carbon-based or porous polymer coating material. These cartridges are essentially functioning as miniature reverse phase (RP) chromatography columns. Adsorption of the analytes onto the sorbent matrix occurs due to Van Der Waal interactions between the carbon-hydrogen bonds of geosmin and 2-MIB and the carbon-hydrogen bonds of the sorbent functional groups. Whereas the silica cartridge contains an active hydrophilic surface, coated with acidic silanol functional groups, which provides a more polar stationary phase somewhat similar to that of a normal phase (NP) chromatography column. Therefore, we anticipated the more polar solvents (methanol, ethanol, and 2-propanol) to be most compatible with the five surface modified silica cartridges and the non-polar n-hexane to elute better with the silica cartridge, as would be expected with conventional RP and NP chromatography, respectively.

Both geosmin and 2-MIB showed a similar extraction performance pattern across each cartridge/solvent combination. Overall, 2-propanol provided the highest elution efficiency for all cartridge types, except for the silica (see Figs. 2 and 3). Both the C18 and hydrophilic C18 cartridges, in combination with 2-propanol elution, provided the best extraction efficiency for geosmin and 2-MIB. However, the hydrophilic cartridge marginally produced a higher extraction efficiency with reduced variation. The hydrophilic C18 μSPE cartridges contain proprietary trimethylsilyl endcapping groups which shield the C18 bonding chains. These modifications avoid the matting-down effect of the C18 chains, whereby the C18 side chains can collapse when using larger aqueous volumes in the mobile phase. Therefore, the hydrophilic C18 cartridges produced a more stable extraction efficiency for both compounds over the conventional C18 cartridges. As a result, for all future method development, the hydrophilic C18 cartridge, with 2-propanol elution solvent, was used.

### Sample loading runs

Table 3 shows repeated sample loading for spiked Type 1 water samples (n = 3). Samples, of 8 mL, were loaded through the hydrophilic C18 cartridge over three consecutive cycles to measure the recoveries of both geosmin and 2-MIB over repeated runs. After running a water sample through the cartridge once, 95.4% and 96.2% of the geosmin and 2-MIB, respectively, was extracted. This shows how highly efficient the cartridges are at binding geosmin and 2-MIB to the hydrophilic C18 surface area. Running a sample through a second and third time recovered only 4.1% and 0.5% of geosmin and 3.6% and 0.2% of 2-MIIB from the water sample, respectively. Therefore, for all future experiments the samples were only loaded once through the cartridges and discarded afterwards.

**Table 3:** Average percentages of geosmin and 2-MIB extracted over three successive sample runs using the hydrophilic C18 μSPE cartridges with 2-propanol elution solvent.

Sample Run	Geosmin	2-MIB
	Average ± SEM (%)	Average ± SEM (%)
1	95.4 ± 0.8	96.2 ± 0.4
2	4.1 ± 0.7	3.6 ± 0.4
3	0.5 ± 0.1	0.2 ± 0.0

### Elution profile

Table 4 shows the elution profile for geosmin, 2-MIB and the internal standard (cis-decahydro-1-napthol), using the hydrophilic C18 cartridge and 2-propanol solvent, from spiked type 1 water samples (n = 3). The elution profile was measured across the first 200 μL, at 50 μL intervals, to identify where the three aforementioned compounds were eluting. By identifying the elution profile for each compound, we could discern the most concentrated part of the eluant to provide the maximum enrichment factor possible. For all compounds the first 50 μL of solvent eluted contained 97.9% and 99.2% of geosmin and 2-MIB, respectively, that was bound to the hydrophilic C18 cartridge. Similarly, the internal standard, cis-decahydro-1-napthol, produced a very similar response to the two analytes with 99.5% being eluted within the first 50 μL when using 2-propanol solvent.

**Table 4:** The percentage of geosmin and 2-MIB eluted by 2-proponal from a hydrophilic C18 μSPE cartridge over the first 200 μL at 50 μL intervals.

Elution volume (μL)	Geosmin	2-MIB	*cis*-Decahydro-1-naphthol
	Average ± SEM (%)	Average ± SEM (%)	Average ± SEM (%)
0 – 50	97.9 ± 0.043	99.2 ± 0.026	99.5 ± 0.037
50 – 100	1.5 ± 0.031	0.5 ± 0.018	0.3 ± 0.030
100 – 150	0.5 ± 0.014	0.2 ± 0.007	0.2 ± 0.003
150 – 200	0.2 ± 0.003	0.1 ± 0.001	0.1 ± 0.007

Therefore, for all further work a 50 μL 2-propanol elution volume was used with the hydrophilic C18 cartridge.

## Analytical performance

### Calibration curves

The hydrophilic C18 μSPE cartridge was used for all performance analysis with 50 μL of 2-propanol elution solvent as optimised in the previous sections. Water samples (n = 3) were spiked with geosmin and 2-MIB to produce final concentrations at 0, 10, 20, 50, 100 and 200 ng L^−1^ for calibration curves (shown in Figs. S3 and S4 in the supporting information). Sample volumes were increased from the 8 mL used in the previous work to 25 mL to achieve sufficient signal levels for both analytes, with odour threshold limits requiring sub 10 ng L^−1^ sensitivity. The 25 mL spiked water samples were finally eluted from the μSPE into 2-propanol following the aforementioned method. Both compounds showed excellent linearity with geosmin at 0.9998 and 2-MIB at 0.9994. Limits of detections (LODs) were calculated using regression analysis based on a 95% confidence interval. LODs were calculated using the formula $LOD=3.3\times \frac{\sigma }{s}$ where $\sigma$ is the standard deviation of the response and $s$ is the slope. The LODs were 2.0 ng L^−1^ and 4.3 ng L^−1^ for geosmin and 2-MIB, respectively. These LODs are comparable to that achieved with traditional SPE pre-conditioning methods seen in Table 1 for geosmin and 2-MIB extraction. Additionally, both analytes are below the 10 ng L^−1^ odour threshold detection levels found within the literature and required for this analysis.

### Cartridge life longevity

The μSPE cartridges used are designed for repeated usage. In a previous study by Alexandrou, et al.^33^ C18 μSPE cartridges were successfully used for 12 successive wastewater samples for THM analysis and showed no change in recovery rates. However, they did note that there was a visible black line that appeared across the top of the sorbent bed. Similarly, Porto-Figueira, et al.^35^ used the PS/DVB μSPE cartridges for more than 40 consecutive extractions of phenolic compounds from teas without losing any performance. However, both Alexandrou, et al.^33^ and Porto-Figueira, et al.^35^ used significantly lower sample volumes (100 μL and 200 μL, respectively) than the 25 mL as required in this study. The performance and longevity of the cartridges were tested by examining repeated sample runs. Water samples were spiked with geosmin and 2-MIB and the extraction efficiency was monitored for 20 repeated 25 mL sample loadings; for both compounds no recovery losses were observed.

### Recoveries and relative standard deviation from raw samples

Raw samples (n = 3) were collected from United Utilities, the water and wastewater providers for the North West of England, for analysis. Specifically, samples were collected from the River Dee at Huntington and Pen-Y-gwely reservoir at Oswestry. The samples were spiked with geosmin and 2-MIB at 10, 50, 100 and 200 ng L^−1^. The extraction procedure described previously was used, with a hydrophilic C18 cartridge, using a 25 mL sample and 50 μL of 2-propanol elution solvent. The recoveries and relative standard deviations (RSD) can be seen in Table 5 below. Relative recoveries were calculated using peak area measured/peak area expected × 100. RSDs were calculated using the formula: $RSD = 100{ \times }\frac{\sigma }{{\overline{x}}}$ where $\sigma$ is the standard deviation of the response and x̄ is the sample mean.

**Table 5:** Mean percentage recoveries of geosmin and 2-MIB from spiked raw water samples extracted using a hydrophilic C18 μSPE cartridge, 2-propanol elution solvent and GC–MS detection.

Water type	Spiked concentration (ng L^−1^)	Geosmin	2-MIB
		Recovery ± RSD (%)	Recovery ± RSD (%)
Oswestry reservoir	Blank	N.D.	N.D.
	10	97.9 ± 3.8	96.3 ± 2.3
	50	98.4 ± 4.4	96.12 ± 4.0
	100	97.8 ± 2.6	98.2 ± 2.1
	200	100.0 ± 2.6	96.0 ± 1.2
Huntington river	Blank	N.D.	N.D.
	10	97.1 ± 2.4	96.6 ± 4.6
	50	97.2 ± 3.0	95.1 ± 7.0
	100	96.9 ± 1.8	95.8 ± 3.4
	200	97.7 ± 1.9	96.7 ± 1.9

*N.D.* not detected.

Table 5 shows excellent recoveries for both analytes from Oswestry reservoir and Huntington River samples. Geosmin recoveries ranged from 96.9 to 100.0% and 2-MIB from 95.1 to 98.2% across all samples and concentrations. RSDs ranged from 1.8 to 4.4% for geosmin and 1.2% to 7% for 2-MIB. These recoveries and RSDs are comparable, and in some instances better, than reports using conventional SPE (Table 1)—yet only a fraction of sample and elution solvents are required, and the process is simple and automated.

## Conclusion

This paper describes a simple, fully automated pre-conditioning step for geosmin and 2-MIB extraction for drinking water analysis using μSPE-GC–MS. The method uses significantly less sample and elution volumes than conventional SPE methods found within the literature, making it more environmentally friendly. Furthermore, it significantly reduces the time-consuming and labour-intensive pre-conditioning steps usually found within the literature and provides a viable method for routine commercial analysis for these two taste and odour compounds. An additional benefit of μSPE is that it is amiable for on-site sampling^36^, enabling rapid screening. If for example a particular water intake was deemed at risk of having high T & O levels, then a suitable portable system would be ideal to make a fast and informed decision regarding resource management. Another scenario of interest is to perform μSPE extraction in-transit. For any water company a single water sampler will often collect dozens of samples per day and deliver them to the lab for subsequent analysis. With a simple and automated pre-conditioning step as described herein, it is possible to complete this process prior to arriving at the lab whilst in-transit—thus allowing the extracted samples (bound within their respective μSPE cartridges) to be simply loaded on to an autosampler for detection (e.g., GC–MS) upon arrival. Such an approach would allow rapid turnaround of results on the same day routinely. This is the subject of future work. The μSPE method demonstrated in this work has potential to be used in any field where classic SPE is required for sample analysis, providing a wide scope for their use in water and, more generally, environmental analysis.

## Supplementary Information

Supplementary Figures.

## Data Availability

The data generated or analysed during this study are presented in the published article and corresponding supplementary information files.
