# Supplementary material for: An automated micro solid phase extraction gas chromatography–mass spectrometry (μSPE-GC–MS) detection method for geosmin and 2-methylisoborneol in drinking water
Source: Sci Rep. 2023 Jan 31;13:1768. doi: 10.1038/s41598-023-28543-x (PMC9889310; doi:10.1038/s41598-023-28543-x)
Supplement: Supplementary file 1 — Supplementary Figures. [file 41598_2023_28543_MOESM1_ESM.pdf]

Supplementary Information

An Automated Micro Solid Phase Extraction Gas Chromatography-Mass Spectrometry (µSPE-GC-MS) Detection Method for Geosmin and 2-Methylisoborneol in Drinking Water

R. L. Bristow [a], A. Haworth-Duff [a], I. S. Young [b], P. Myers [c], M. R. Hampson [d], J. Williams [d], S. Maher [a*]

a) Department of Electrical Engineering and Electronics, University of Liverpool, Liverpool, United Kingdom.

b) Institute of Life Course and Medical Sciences, University of Liverpool, Liverpool, United Kingdom.

c) Department of Chemistry, University of Liverpool, Liverpool, United Kingdom.

d) United Utilities, Lingley Mere Business Park, Warrington, United Kingdom.

* For correspondence, email: s.maher@liverpool.ac.uk

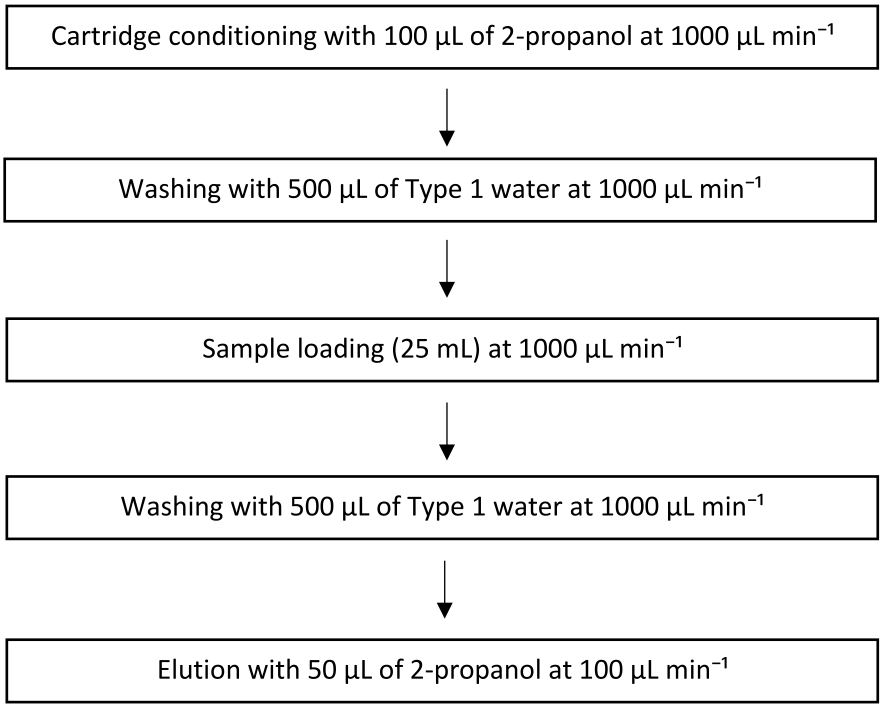

Figure S1: Overview of the operational stages for µSPE sample preparation method used in this study.

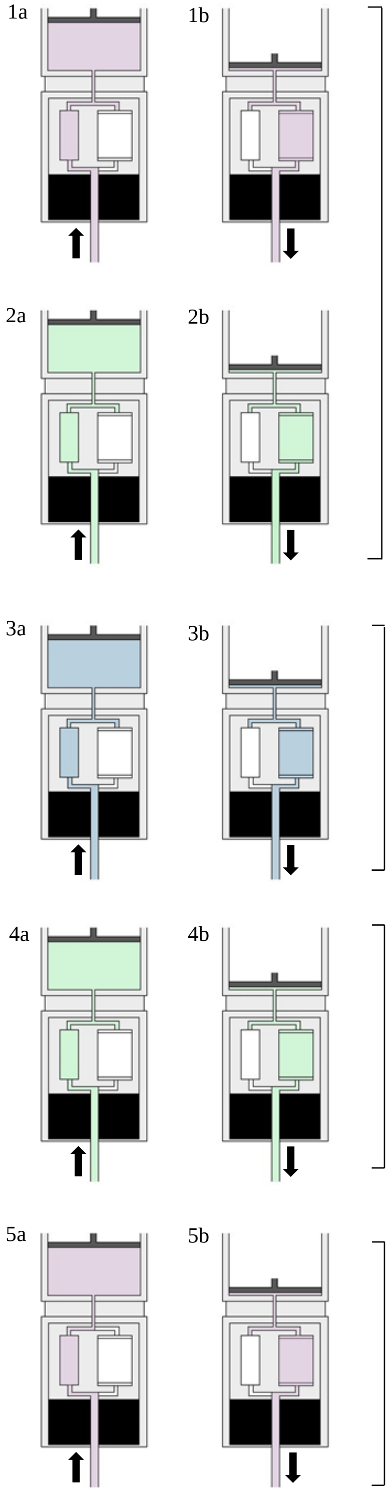

Conditioning

Firstly, the µSPE cartridge is conditioned using 2-propanol solvent to remove any prior bound analytes from previous extractions. 100 µL of 2-propanol is aspirated (1a) and dispensed (1b) at 1000 µL min^{-1}.

Secondly, ultrapure type 1 water is used to remove any residual solvent. 500 µL of ultrapure water is aspirated (2a) and dispensed (2b) at 1000 µL min^{-1}.

Loading

A 25 mL water sample is aspirated (3a) at 1000 µL min^{-1} and dispensed (3b) at 500 µL min^{-1}.

Washing

500 µL of ultrapure type 1 water is aspirated (4a) and dispensed (4b) at 1000 µL min^{-1}. This removes any unbound material from the hydrophilic C18 bed surface, leaving only bound analytes.

Elution

100 µL of 2-propanol solvent is aspirated (5a) at 1000 µL min^{-1} and the first 50 µL is dispensed (5b) at 100 µL min^{-1} into the autosampler vial eluting the bound T&O analytes from the cartridge matrix for analysis. The remaining 50 µL of elution solvent is discarded.

Figure S2: Illustrates the aspirations (a) and dispensations (b) on the µSPE cartridge/syringe driver interface and details of the four main stages – conditioning (1a/1b & 2a/2b), loading (3a/3b), washing (4a/4b) and elution (5a/5b) – for the µSPE extraction method described in the corresponding manuscript.

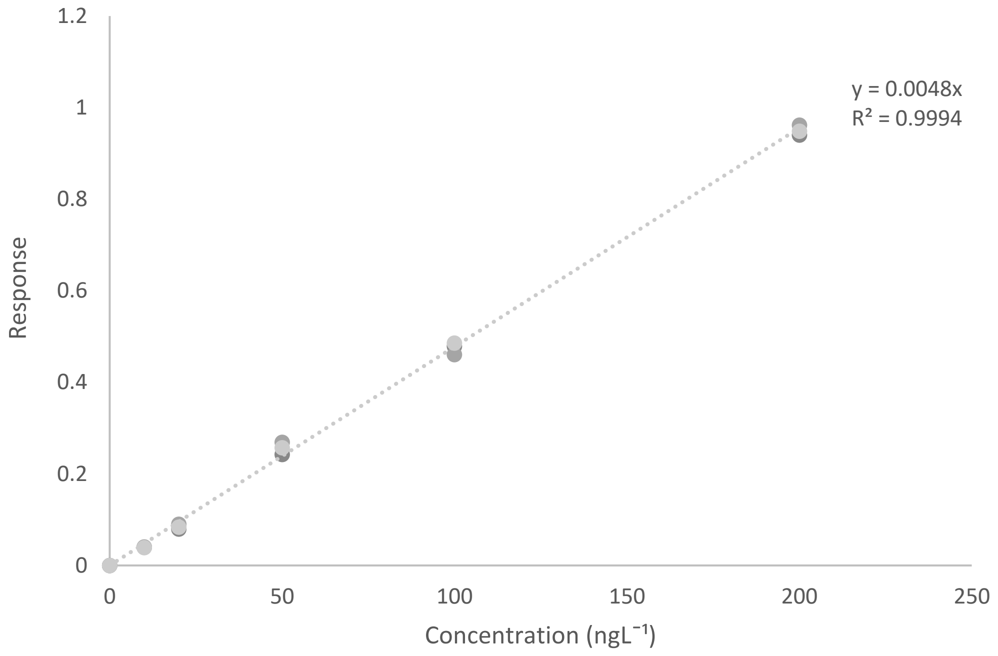

Figure S3: Calibration curve for 2-MIB spiked at concentrations of 0, 10, 20, 50, 100 and 200 ngL^{-1} into ultrapure Type 1 water.

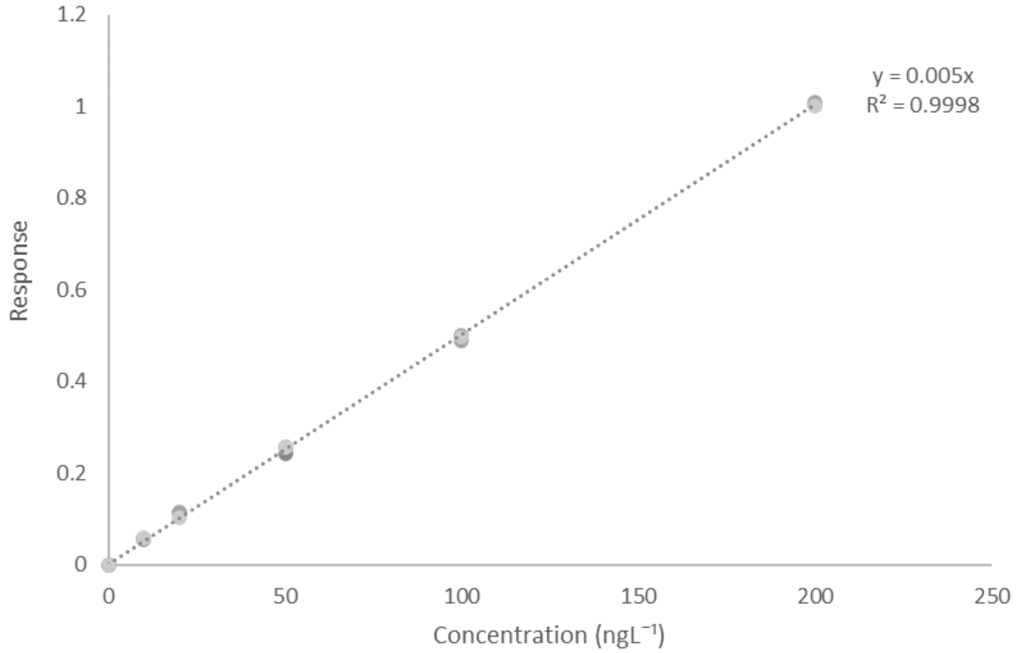

Figure S4: Calibration curve for geosmin spiked at concentrations of 0, 10, 20, 50, 100 and 200 ngL^{-1} into ultrapure Type 1 water.
